# Evaluation of LLM-generated peptide as foundation template for discovery of effective encrypted AMPs against clinical superbugs

**DOI:** 10.1128/spectrum.01504-25

**Published:** 2025-09-02

**Authors:** Lanlan Zhao, Yihui Wang, Jun Jiang, Hongwei Pan, Lijuan Wang, Shutao Ma, Lei Zhang

**Affiliations:** 1 Microbiome-X, School of Public Health, Cheeloo College of Medicine, Shandong University, Jinan, China; 2 Department of Medicinal Chemistry, Key Laboratory of Chemical Biology (Ministry of Education), School of Pharmaceutical Sciences, Cheeloo College of Medicine Shandong University, Jinan, China; 3 Department of Clinical Laboratory, Qilu Hospital of Shandong University 91623, Jinan, China; 4 Neck-Shoulder and Lumbocrural Pain Hospital of Shandong First Medical University, Shandong First Medical University, Shandong Academy of Medical Sciences, Jinan, China; 5 State Key Laboratory of Microbial Technology, Shandong University, Qingdao, China; Houston Methodist, Houston, Texas, USA

**Keywords:** antimicrobial peptides, generative model, sequence alignment, multidrug-resistant, CRAB

## Abstract

Novel antimicrobial agents are urgently needed to combat the antibiotic-resistance crisis, particularly in the face of multidrug-resistant (MDR) pathogens like carbapenem-resistant *Acinetobacter baumannii* (CRAB) and methicillin-resistant *Staphylococcus aureus* (MRSA). In this study, we present an approach that combines generative large language model with sequence alignment to identify promising antimicrobial peptides. With this strategy, we rapidly identified five novel encrypted peptides based on a generated template, demonstrating significant antimicrobial activity against a broad spectrum of clinical MDR pathogens. Among them, PL-15 stood out as a potent, broad-spectrum AMP with comparable therapeutic efficacy to polymyxin B against CRAB infections *in vivo*. Mechanistic investigations revealed that PL-15 exerts its bactericidal effects by disrupting both the outer and cytoplasmic membranes, causing membrane depolarization, elevating intracellular reactive oxygen species (ROS) levels, and ultimately leading to rapid bacterial cell death. Additionally, PL-15 demonstrated remarkable antibiofilm activity, inhibiting biofilm formation and eradicating pre-existing biofilms, further reducing the risk of resistance development. This work highlights the potential of using generative model combined with sequence alignment to accelerate the discovery of novel analogs with enhanced properties.

**IMPORTANCE:**

The rise of multidrug-resistant pathogens, such as carbapenem-resistant *Acinetobacter baumannii* and methicillin-resistant *Staphylococcus aureus*, poses a severe threat to public health, making the search for novel antimicrobial agents a critical priority. In this study, we present an innovative approach combining generative large language models and sequence alignment to identify promising antimicrobial peptides. This method allowed for the rapid discovery of five encrypted peptides with strong antimicrobial activity against a range of multidrug-resistant pathogens. Among them, PL-15 showed remarkable efficacy, comparable to polymyxin B, and exhibited potent antibiofilm properties, making it a strong candidate for further development. By providing a novel approach to discovering antimicrobial agents, this work presents a promising solution to the escalating crisis of antibiotic resistance.

## INTRODUCTION

The global crisis of multidrug-resistant (MDR) pathogens is driving growing concerns about the emergence of untreatable infections, which are responsible for a substantial number of patient deaths (1). The World Health Organization (WHO) has highlighted a group of high-priority MDR pathogens, collectively referred to as ESKAPE, which includes *Enterococcus faecium*, *Staphylococcus aureus*, *Klebsiella pneumoniae*, *Acinetobacter baumannii*, *Pseudomonas aeruginosa*, and *Enterobacter* species (2). Among these, carbapenem-resistant *A. baumannii* (CRAB) is of particular concern due to its role as a leading cause of hospital-acquired infections, including bacteremia, urinary tract infections, and ventilator-associated pneumonia (3). The increasing prevalence of CRAB, particularly in intensive care settings, underscores the urgent need for new antimicrobial agents to combat these deadly pathogens.

In recent years, large language models (LLMs) have revolutionized the field of natural language understanding, with significant advances in artificial general intelligence. Within the biological sciences, specialized LLMs have been developed to address unique challenges across various subdomains. Molecular LLMs, for example, are used to predict molecular properties and design novel drugs (4, 5), while protein-focused models assist in predicting protein structures and engineering proteins with desired functionalities (6–10). Genomic LLMs facilitate the annotation of gene functions and the analysis of sequence variations and evolutionary patterns (11–13). These applications demonstrate the growing importance of LLMs in the biological sciences, enabling more efficient solutions to complex problems.

In our previous work (14), we developed a universal protein sequence LLM, ProteoGPT, and further fine-tuned it for AMP generation through transfer learning, resulting in the creation of AMPGenix. The transfer learning approach endowed AMPGenix with an enhanced understanding of the amino acid distribution, composition, and tendencies characteristic of AMP sequences. Peptides generated by AMPGenix exhibited a potential bacterial inhibitory rate of 61.90%. Building on this success, we hypothesized that these sequences could serve as versatile templates for the design and modification of AMPs. Previous study (15) had emphasized the proteome as an untapped resource for discovering antimicrobial agents, with increasing evidence suggesting that some AMPs are encoded within proteins in an encrypted form. These encrypted peptides, concealed within the broader sequence of functional proteins, may represent a novel class of antimicrobial molecules that could be harnessed for drug development. In our study, we employed an approach combining AMP generative model with sequence alignment and rapidly identified five novel analogs exhibiting high similarity to the template. These encrypted peptides, embedded within membrane-associated proteins from various microbes, demonstrated significant antimicrobial activity against a range of clinical MDR superbugs, including CRAB and methicillin-resistant *S. aureus* (MRSA). Among these, PL-15 is a superior broad-spectrum AMP against both gram-positive and gram-negative bacteria, including drug-sensitive and drug-resistant strains. It demonstrates comparable therapeutic efficacy against CRAB infections *in vivo* compared to polymyxin B. Mechanism of action (MoA) experiments revealed that PL-15 exerts its bactericidal effects by increasing outer membrane permeability, disrupting cytoplasmic membrane integrity, inducing membrane depolarization, and elevating intracellular reactive oxygen species (ROS) levels, which collectively contribute to rapid bacterial cell death. Notably, PL-15 exhibits more rapid bactericidal activity than clinical antibiotics while simultaneously inhibiting biofilm formation and eradicating pre-existing mature biofilms. This comprehensive antimicrobial action results in significantly reduced potential for resistance development.

## RESULTS

### The sources and structural characteristics of c_AMPs

**Fig 1:**
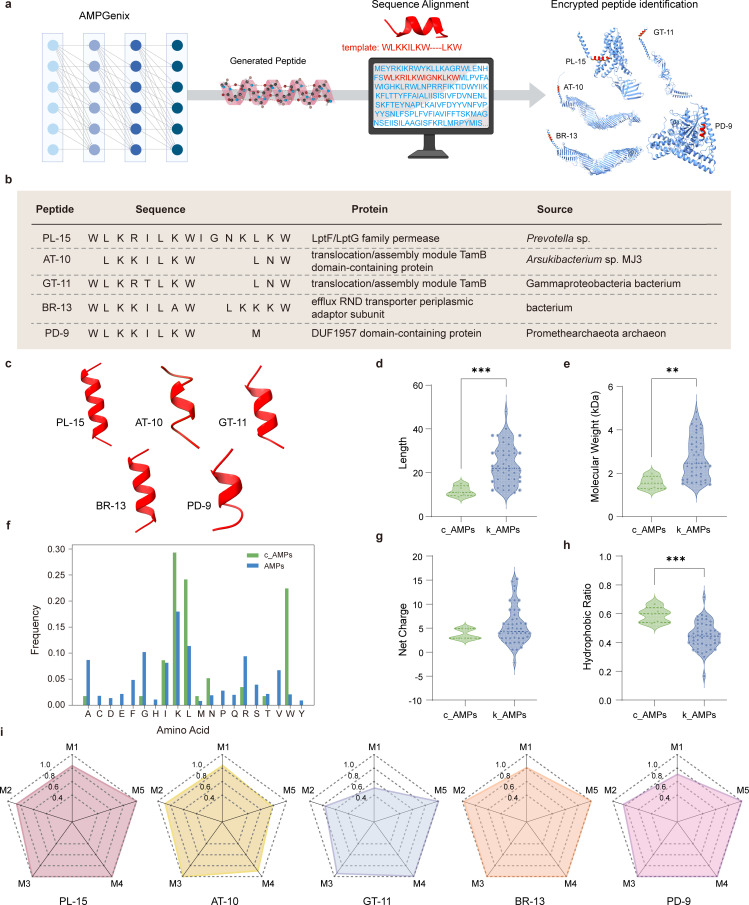
The discovery and characteristic statistics of novel encrypted peptides. (**a**) Pepline combining generative model with sequence alignment for encrypted peptide identification. (**b**) Description of the results via sequence alignment. (**c**) The secondary structures of five c_AMPs predicted by AlphaFold. (**d and e**) Comparison of sequence length (**d**) and molecular weight (**e**) between c_AMPs and 47 k_AMPs. ^*^P < 0.05; ^**^P < 0.01; ^***^P < 0.005; Mann-Whitney *U* test. (**f**) Amino acid composition of c_AMPs compared with known AMPs collected. (**g and h**) Comparison of net charge in pH 7.4 (**g**) and hydrophobic ratio (**h**) between c_AMPs and k_AMPs. ^*^P < 0.05; ^**^P < 0.01; ^***^P < 0.005; Mann-Whitney *U* test. (i) The predicted probabilities of c_AMPs under different AMP classification models. (M1: Macrel; M2: AmPEP; M3: AMP Scanner V2; M4: AMPlify_bal; M5: AMPlify_imbal). The schematic in (**a**) was created using BioRender.com.

In previous studies, we discovered that the short peptide g_AMP41 (WLKKILKWLKW), generated by AMPGenix, exhibited excellent antimicrobial activity against MRSA (minimum inhibitory concentration [MIC] = 8 ~ 13.33 µg mL^−1^) and CRAB (MIC = 8 µg mL^−1^) *in vitro* but showed suboptimal efficacy in treating infections *in vivo*, leaving room for further modification (14). Using this validated AMP as a template, we performed sequence alignment with the National Center for Biotechnology Information (NCBI) database to identify encrypted AMPs. Five protein segments with highest Max Score were identified as candidate AMPs (c_AMPs) (Fig. 1a; Table S1). A retrospective analysis of their precursor proteins was conducted to understand their biological context using NCBI annotations. The results showed that these c_AMPs (PL-15, AT-10, GT-11, BR-13, and PD-9) were derived from five distinct microbial species, each contributing uniquely to ecological and physiological processes (Fig. 1b). Specifically, PL-15 is sourced from *Prevotella* sp., a genus of gram-negative bacteria that plays a critical role in the human microbiome, particularly within the oral and gastrointestinal tract environments (16). AT-10 originates from *Arsukibacterium* sp. MJ3 and was isolated from cold and alkaline environment as producers of extracellular proteolytic enzymes active at high pH and low temperature (17). GT-11 is derived from Gammaproteobacteria, a large and diverse class of bacteria that encompasses a variety of pathogens as well as environmental microorganisms (18). BR-13 is obtained from an unidentified bacterium within the Resistance-Nodulation-Cell Division transporter family, which is notable for its involvement in bacterial drug resistance mechanisms (19). PD-9, derived from the archaeon Promethearchaeota, is especially intriguing due to its potential resilience to extreme environmental conditions, a characteristic typical of archaea (20).

These peptides exhibit sequence identities ranging from 60% to 90%, revealing a significant degree of sequence homology across these diverse microbial origins. This similarity suggests that despite their varied microbial sources, the peptides may share common functional mechanisms. Notably, these peptides are associated with membrane-related proteins involved in essential processes such as transport, assembly, and efflux, particularly within protein complexes that govern translocation and efflux systems. This functional linkage lends support to the hypothesis that structural compatibility helps these c_AMPs penetrate into bacterial cells and exert antibacterial effects.

To comprehensively evaluate the five c_AMPs, we compared them with 47 known natural AMPs (k_AMPs, Table S2) previously reported with anti-*A*. *baumannii* activity (21–46), identified through a literature search. Similar to most known AMPs, the five c_AMPs also exhibit an α-helical structure (Fig. 1c). However, the results revealed that c_AMPs are significantly shorter, with lengths ranging from nine to 15 amino acids and an average length of 11.6. In contrast, the k_AMPs exhibit a broader length range, from 11 to 48 amino acids, with an average of 23.9 (Fig. 1d). Furthermore, the average molecular weight of c_AMPs is 1570.99 Da, approximately 40% lower than that of the k_AMPs, which have an average molecular weight of 2693.02 Da (Fig. 1e). The shorter length and reduced molecular weight of c_AMPs likely contribute to their enhanced absorption and stability in biological systems.

Additionally, the amino acid composition of c_AMPs shows significant differences compared to AMPs (Fig. 1f). Specifically, c_AMPs exhibit a higher enrichment of certain amino acids, which play a critical role in the antimicrobial activity of these peptides. Lysine (Lys, K), a common positively charged amino acid, is present at a higher frequency in c_AMPs, which contributes to their overall strong positive charge (average net charge = 3.7) (Fig. 1g). This positive charge facilitates effective interaction with the negatively charged bacterial membranes, enhancing their membrane-disrupting ability. Moreover, the enrichment of hydrophobic amino acids like leucine (Leu, L), isoleucine (Ile, I), and tryptophan (Trp, W) leads to a higher hydrophobic ratio in c_AMPs (Fig. 1h), improving their affinity for bacterial membranes. Hydrophobic amino acids promote the binding of the peptides to the bacterial membrane, disrupting its integrity, thereby boosting their antimicrobial efficacy. In particular, Trp, an aromatic amino acid with strong hydrophobicity, not only enhances the interaction between the peptide and the membrane but also improves the structural stability of the peptide, making it more resilient under various environmental conditions.

The amino acid composition of c_AMPs suggested a strong potential for antimicrobial activity, which is further supported by their performance in various AMP classification models. Indeed, the five c_AMPs were all predicted as AMP with high probabilities across five different models (Fig. 1i), reinforcing the hypothesis that their distinctive structural features contribute to their potent antimicrobial properties.

### PL-15 exhibits broad-spectrum and superior antibacterial activity against MDR superbugs

**Fig 2:**
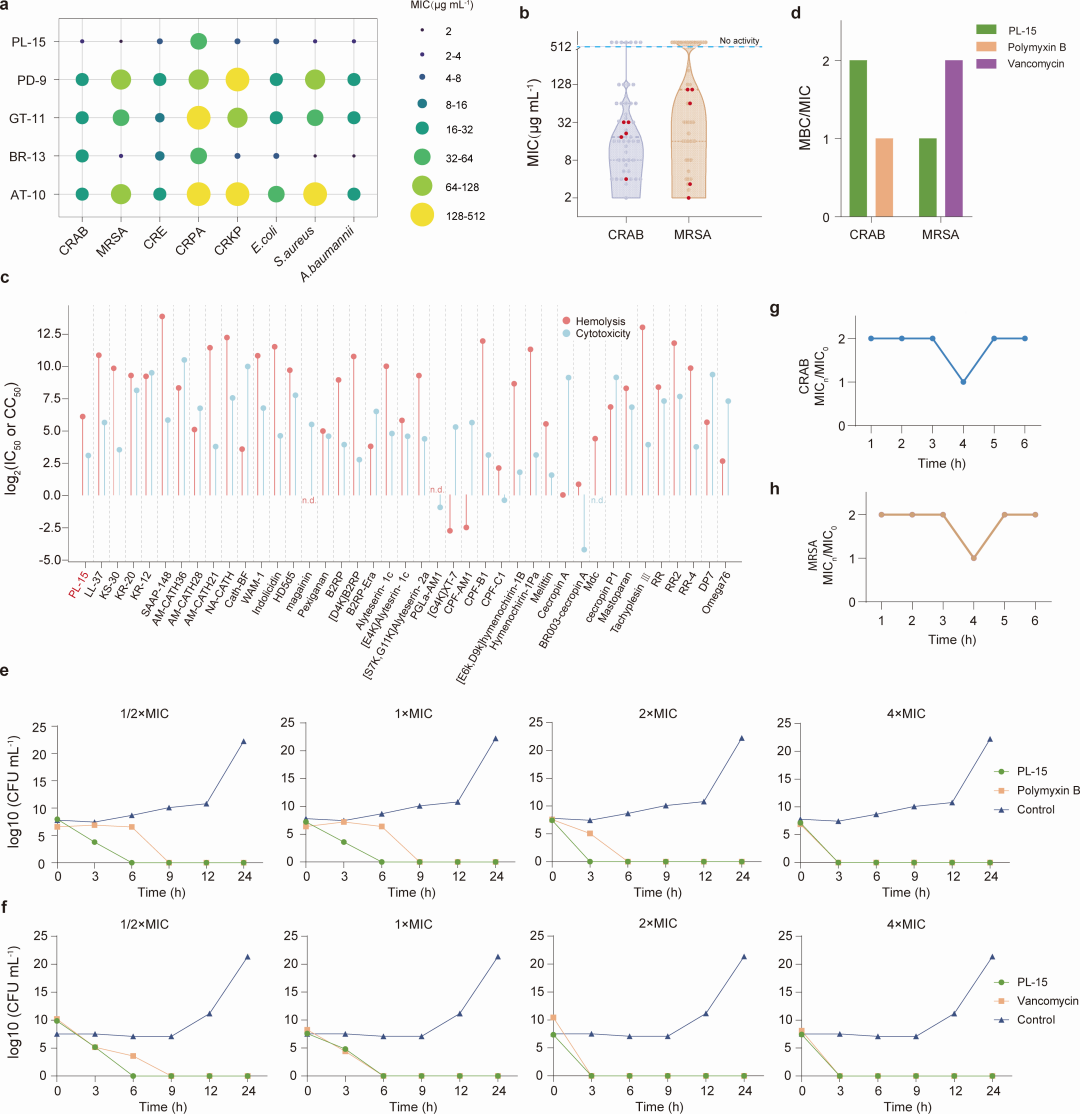
Evaluation of antibacterial and bactericidal effect and toxicity *in vitro*. (**a**) Antibacterial activity levels of five c_AMPs against *E. coli* ATCC25922, *S. aureus* ATCC25923, *A. baumannii* ATCC19606, and ICU-isolated pathogenic bacterial strains (CRAB 2210186, MRSA 2210187, CRE 2403262, CRPA 2403282, and CRKP 2403276). The bubble size and color indicate the MIC values. (**b**) MIC values of the c_AMPs and k_AMPs against CRAB and MRSA assayed under unified experimental standards. The dots marked in red in the figure are c_AMPs. (**c**) Hemolysis and cytotoxicity assays determined IC50/CC50 for PL-15 and 40 k_AMPs with antimicrobial activity against both CRAB and MRSA, shown as log_2_-transformed values. n.d., not determined—that is, no hemolytic or cytotoxic effects. (**d**) MBC/MIC ratio of PL-15 and polymyxin B or vancomycin against CRAB and MRSA. €, time-kill kinetics of PL-15 and polymyxin B against CRAB. (**f**) Time-kill kinetics of PL-15 and vancomycin against MRSA. (**g and h**) The ratio of the MIC value of PL-15 to CRAB (**g**) and MRSA (**h**) after co-treatment with fetal bovine serum for different periods of time to the initial MIC value.

The antimicrobial activities of chemically synthesized c_AMPs were systematically assessed against a panel of gram-positive and gram-negative bacterial strains, including both drug-sensitive (*Escherichia coli* ATCC25922, *S. aureus* ATCC25923, and *A. baumannii* ATCC19606) and drug-resistant phenotypes (CRAB 2210186, MRSA 2210187, carbapenem-resistant *Enterobacterales* [CRE] 2403262, carbapenem-resistant *P. aeruginosa* [CRPA] 2403282, and carbapenem-resistant *K. pneumoniae* [CRKP] 2403276). The results showed that these five c_AMPs exhibited antimicrobial activity against all eight bacterial strains (MIC ≤5 12 µg mL^−1^, Fig. 2a). MIC values for sensitive bacteria varied, with *E. coli* ranging from 8 to 42.67 µg mL^−1^, *A. baumannii* from 1 to 32 µg mL^−1^, and *S. aureus* from 4 to 256 µg mL^−1^. When tested against clinical drug-resistant bacteria, the five c_AMPs demonstrated stronger antibacterial effects against CRAB, MRSA, and CRE. The MIC values for CRAB ranged from 4 to 32 µg mL^−1^, for MRSA from 2 to 106.67 µg mL^−1^, for CRE from 8 to 21.33 µg mL^−1^, for CRPA from 64 to 426.67 µg mL^−1^, and for CRKP from 8 to 256 µg mL^−1^. Notably, PL-15 and BR-13 demonstrated broad-spectrum antimicrobial properties against both gram-negative and gram-positive bacteria, with MICs for all tested bacteria ≤64 µg mL^−1^. In particular, against CRAB and MRSA, PL-15 and BR-13 showed the strongest inhibitory effects (MIC_CRAB_PL-15_ = 4 μg mL^−1^, MIC_MRSA_PL-15_ = 2 μg mL^−1^, MIC_CRAB_BR-13_ = 18.67 μg mL^−1^, and MIC_MRSA_BR-13_ = 3.33 μg mL^−1^). Additionally, these two peptides had MICs of 64 µg mL^−1^ against CRPA and MICs lower than 11 µg mL^−1^ against CRE, CRKP, and three sensitive strains. In comparison, AT-10, GT-11, and PD-9 displayed a narrower antimicrobial spectrum, demonstrating higher sensitivity to CRE than other MDR bacteria.

To assess the relative activity of c_AMPs, we evaluated inhibitory activity of 47 k_AMPs against CRAB and MRSA under uniform experimental conditions (Fig. 2b). The results showed that 40 out of 47 k_AMPs exhibited significant antimicrobial activity (MIC ≤ 128 µg mL^−1^) against CRAB, and seven were ineffective with MIC > 512 µg mL^−1^. A total of 24 k_AMPs showed MIC values ≤ 128 µg mL^−1^ against MRSA, and 16 were ineffective. Overall, only 24 k_AMPs exhibited MIC values ≤ 128 µg mL^−1^ against both CRAB and MRSA, whereas all five c_AMPs showed equally effective antimicrobial activity against both MDR pathogens, demonstrating superior overall effects (Fig. 2b).

Notably, PL-15 exhibited the most potent antimicrobial activity, surpassing the eight top-performing k_AMPs (B2RP-Era, [S7K,G11K]alyteserin-2a, [D4K]B2RP, [G4K]XT-7, pexiganan, [E6k,D9k]hymenochirin-1B, CPF-B1, and hymenochirin-1Pa), all of which demonstrated MICs below 8 µg mL^−1^ against both CRAB and MRSA. Based on these findings, PL-15 was selected as the optimal c_AMP for further evaluation of its cytotoxicity, bactericidal effects, therapeutic efficacy *in vivo*, and underlying antimicrobial mechanisms.

### PL-15 exhibits a favorable toxicity profile

Next, we conducted a comprehensive toxicity evaluation of PL-15 against eukaryotic cells, encompassing both fresh sheep red blood cells and HEK293 cells, and compared its effects with 40 k_AMPs that exhibited antibacterial activity (MIC ≤ 512 µg mL^−1^ for at least one bacterium) under standardized experimental conditions. Hemolysis and cytotoxicity assays were performed at various concentrations, and the respective 50% hemolytic concentration (IC50, 50% hemolysis) and 50% inhibiting concentration (CC50, 50% cell viability) toxicity values were determined (Fig. 2c). The results demonstrated that a total of 35 AMPs (including PL-15) exhibited log_2_IC50 ≥ 3, and 35 AMPs (including PL-15) showed log_2_CC50 ≥ 3. Of these, 31 AMPs (including PL-15) simultaneously met both criteria of log_2_IC50 ≥ 3 and log_2_CC50 ≥ 3. The cytotoxicity profile of PL-15 was comparable to that of four k_AMPs ([E6k,D9k]hymenochirin-1B, [D4K]B2RP, CPF-B1, hymenochirin-1Pa), which displayed antimicrobial activity approaching, though slightly inferior to, that of PL-15. These findings indicate that PL-15 exhibits relatively low hemolysis and cytotoxicity, positioning it within the intermediate range of k_AMPs performance levels.

### PL-15 exhibits rapid and potent bactericidal activity

Bactericides have the advantages of reducing the risk of drug resistance, reducing bacterial spread, and providing more thorough treatment (47). Generally, an minimum bactericidal concentration (MBC)/MIC ratio of ≤4 is considered a bactericidal agent, while an MBC/MIC ratio of >4 is considered an antibacterial agent (48). The MBCs of PL-15 against CRAB and MRSA were determined, with polymyxin B and vancomycin as control, and the MBC/MIC ratios were calculated. The results demonstrated that PL-15 exhibited bactericidal effects on both resistant bacterial strains (Fig. 2d).

Subsequently, a bactericidal kinetic experiment was conducted to investigate the time- and concentration-dependent bactericidal effect of PL-15. As shown in Fig. 2e, at concentrations of 1/2 × and 1 × MIC, PL-15 had a exhibited a faster bactericidal rate than positive control polymyxin B, successfully killing CRAB within 6 h. At 2 × MIC, both PL-15 and polymyxin B showed enhanced bactericidal kinetics for CRAB, with PL-15 achieving complete bacterial eradication within 3 h, compared to polymyxin B’s 6-hour requirement. At 4 × MIC, PL-15 and polymyxin B reached the same bactericidal effect, killing CRAB within 3 h. For MRSA, at 1/2 × MIC, PL-15 demonstrated a more rapid bactericidal effect compared to the positive control vancomycin, effectively eliminating the bacteria within 6 h (Fig. 2f). At 1 × MIC, PL-15 and vancomycin exhibited the same bactericidal effect, both achieving complete bacterial eradication within 6 h. At 2 × and 4 × MIC concentrations, PL-15 and vancomycin demonstrated enhanced efficacy, achieving full bacterial killing within 3 h. In conclusion, PL-15 demonstrates superior bactericidal efficiency at lower concentrations compared to conventional antibiotics, which require higher concentrations to achieve comparable killing effects.

### PL-15 exhibits stable antibacterial activity in serum

The complex composition of serum, particularly the serum proteins, enzymes, and various ions, has been identified as one of the primary factors contributing to the reduced antimicrobial activity of AMPs. These serum components may interact with AMPs, altering their structure or competing for binding sites, which can influence their interaction with bacterial membranes, thus affecting their antimicrobial efficacy (49, 50). To assess the serum sensitivity of PL-15, we examined the fold changes in MIC values for CRAB and MRSA. As shown in Fig. 2g and h, after incubation for 1 to 6 h, PL-15 exhibited a maximum twofold increase in MIC values for both MDR pathogens. These findings suggest that PL-15 retains relatively stable activity in a serum environment, offering promising implications for further *in vivo* evaluation.

**PL-15 demonstrates significant therapeutic efficacy against CRAB** infections *in vivo*

**Fig 3:**
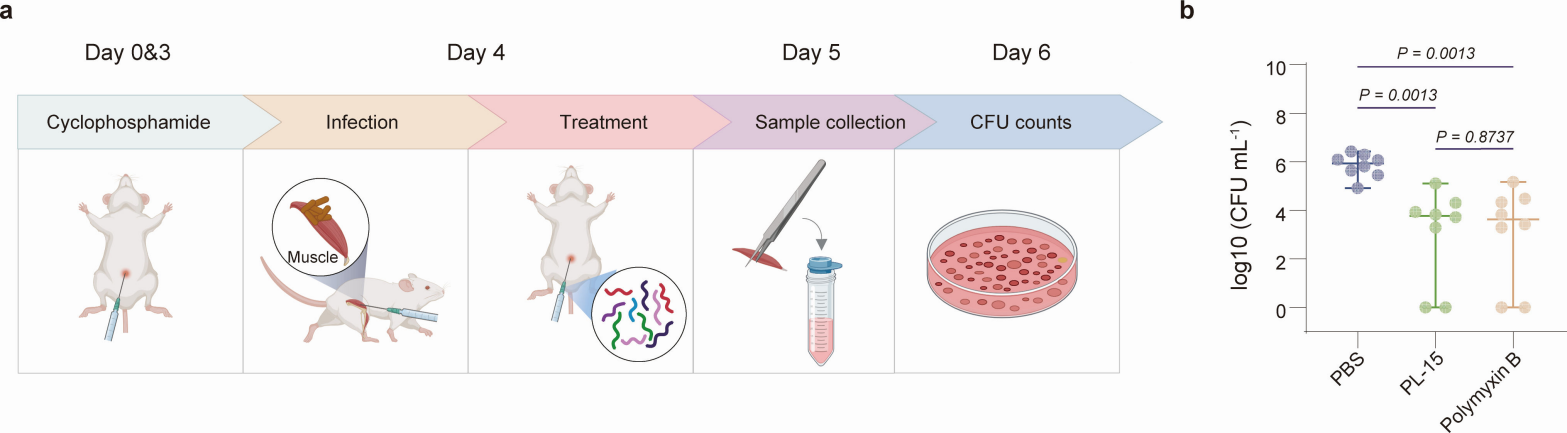
Therapeutic efficiency in treating thigh infections *in vivo*. (**a**) Schematic of thigh infection mouse model to assess the anti-infective activity of PL-15 with activity against CRAB cells. Each mouse was injected with approximately 10^6^ CFU of CRAB in the thigh tissue, followed by four intraperitoneal injections of compounds within 24 h of infection. (**b**) Bacterial loads in different treatment groups, shown as log_10_-transformed values. Each group consisted of eight mice (*n* = 8). The *P*-value was calculated based on the statistical analysis of bacterial counting values. Statistical analysis was conducted using Mann-Whitney *U* test. Schematic in panel **a **was created with BioRender.com.

The WHO has classified CRAB as a “Critical Priority” pathogen in its Priority Pathogens List, recognizing it as a predominant causative agent of hospital-acquired infections (2). This classification reflects CRAB’s global dissemination, high mortality rates, and extreme resistance profiles. Given this critical situation, a thigh infection model was employed to evaluate the *in vivo* therapeutic efficacy of PL-15 against CRAB. To establish the mouse thigh infection model, mice were pre-treated intraperitoneally with cyclophosphamide 4 days and 1 day prior to bacterial inoculation to induce neutropenia, administered at doses of 150 mg kg^−1^ and 100 mg kg^−1^, respectively (51). Each mouse was then injected with approximately 10^6^ colony-forming unit (CFU) of CRAB in the thigh tissue, followed by four intraperitoneal injections of PL-15, polymyxin B, or phosphate-buffered saline (PBS) within 24 h of infection (Fig. 3a).

The results confirmed successful establishment of the infection model, with the PBS control group showing mean bacterial loads of~10⁶ CFU. Both PL-15 and polymyxin B significantly reduced bacterial loads by two orders of magnitude compared to the PBS control group, demonstrating comparable therapeutic efficacy. Both treatments eradicated over 97% of bacteria in the thigh tissue within 24 h (PL-15, *P* = 0.0013; polymyxin B, *P* = 0.0013) (Fig. 3b).

### PL-15 can disrupt bacterial cell membranes

**Fig 4:**
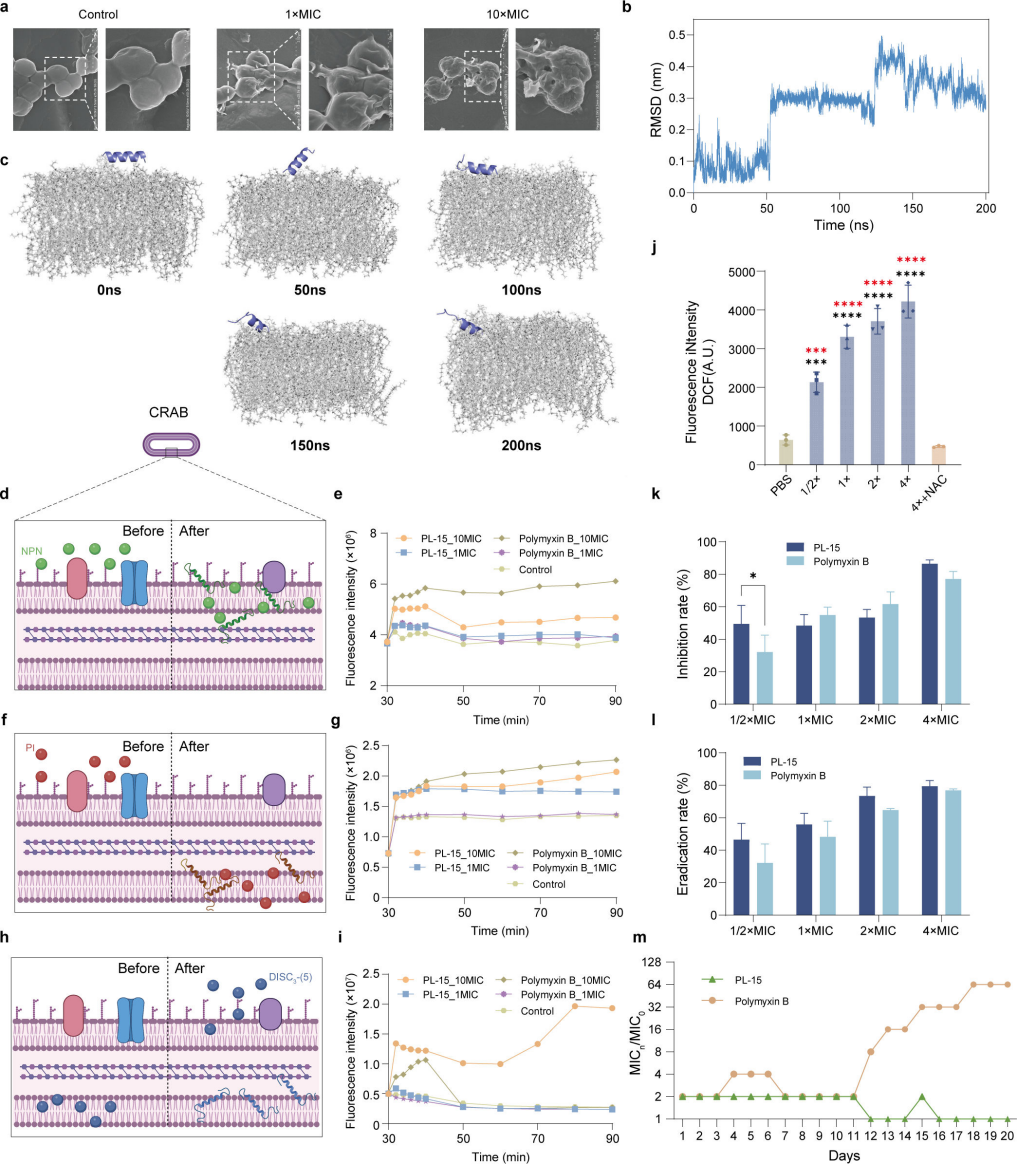
MoA of PL-15. (**a**) The SEM images of CRAB treated with PL-15 and polymyxin B at concentrations of 1 × MIC and 10 × MIC. The control group was untreated corresponding strains. (**b**) RMSD during MD simulations with POPE/POPG. (**c**) System snapshots of PL-15 during MD simulations with POPE/POPG. (**d**) Schematic showing the behavior of NPN, the fluorescent probe used to indicate outer membrane permeabilization caused by PL-15. (**e**) The real-time fluorescence intensity of NPN after adding PL-15 or polymyxin B with 10 × and 1 × MIC. (**f**) Schematic showing the behavior of PI, the fluorescent probe used to show cell membrane disruption caused by PL-15. (**g**) The real-time fluorescence intensity of PI after adding Pl-15 or polymyxin B with 10 × and 1 × MIC. (**h**) Schematic of DiSC_3_- (5), a hydrophobic fluorescent probe, used to show depolarization of cytoplasmic membranes induced by PL-15. (**i**) The real-time fluorescence intensity of DiSC_3_- (5) after adding Pl-15 or polymyxin B with 10 × and 1 × MIC. The untreated wells (buffer + bacteria + fluorescent dye) were used as control. All experiments were performed in triplicate. (**j**) The real-time fluorescence intensity of DCF after adding PL-15 to CRAB. The charts show the mean  ±  s.d. of three independent experiments. All statistically significant differences between samples were tested with one-way analysis of variance. *P < 0.05, **P < 0.01, ***P < 0.001, ****P < 0.0001. Black *: compared with the PBS group, red *: compared with the NAC group. (**k and l**) Biofilm inhibition ability (**k**) and biofilm eradication ability (**l**) of PL-15 and polymyxin B against CRAB. Statistical analysis was conducted using Mann-Whitney *U* test. ^*^P < 0.05. (**m**) Resistance development of CRAB to PL-15 or polymyxin B under 0.5-fold MIC concentrations (*n* = 3 biologically independent replicates). Schematic in (**d**), (**f**), and (**h**) was created with BioRender.com.

The MoA of AMPs differs significantly from that of conventional antibiotics. AMPs primarily target bacterial membranes, inducing membrane rupture and subsequent leakage of cellular contents, ultimately leading to bacterial death. To investigate the impact of PL-15 on bacterial membranes, we visualized the morphological changes of bacterial cells using scanning electron microscopy (SEM) (Fig. 4a). Compared to the control group, SEM images revealed that CRAB treated with PL-15 displayed various degrees of shrinkage, indentation, and irregular surface deformation. Notably, at a concentration of 10 × MIC, bacterial cells showed clear signs of membrane rupture, with visible pores and cracks on their surfaces. These observations demonstrate a dose-dependent effect of AMPs on bacterial cell integrity, further supporting their MoA.

To gain mechanistic insights into the membrane interaction of PL-15, we performed an all-atom molecular dynamics (MD) simulation of PL-15 in the presence of a 1-palmitoyl-2-oleoyl-sn-glycero-3-phosphoethanolamine (POPE)/1-palmitoyl-2-oleoyl-sn-glycero-3-(phospho-rac-(1-glycerol)) (POPG) lipid bilayer. As shown in Fig. 4b and c, PL-15 initially exhibited no stable binding during the first 130 ns, suggesting dynamic sampling of optimal membrane-binding orientations. Around 130 ns, a notable increase in root mean square deviation (RMSD) was observed, indicating conformational rearrangement. Structural analysis revealed that at 150 ns and 200 ns, the N-terminal residues Trp, Ile, and Lys partially inserted into the membrane surface, forming stable interactions. During the simulation, PL-15 maintained its overall α-helical structure, with partial unfolding at the C-terminus after membrane association, potentially reflecting insertion-induced conformational changes. These results suggest that PL-15 may exert its bactericidal activity through interactions with bacterial membranes.

To further investigate clues about the MoA of PL-15, we then conducted fluorescence assays to determine if they act by targeting the membrane. Initially, the impact of PL-15 on bacterial outer membrane permeability was evaluated using a hydrophobic fluorescence dye 1-(N-phenylamino)naphthalene (NPN). In an aqueous solution, NPN does not exhibit any fluorescence and is unable to penetrate the bacterial outer membrane to access the cytoplasm. However, upon alteration of the permeability of the bacterial outer membrane by AMP, NPN gains entry into the cytoplasm and elicits a robust fluorescent response (Fig. 4d). In a manner similar to polymyxin B, PL-15 exhibited only a marginal increase in fluorescence intensity at the lower concentration of 1 × MIC, with fluorescence levels slightly higher than the negative control. However, at a higher concentration of 10 × MIC, PL-15 induced a rapid and significant increase in fluorescence intensity, akin to the response observed with polymyxin B (Fig. 4e).

Next, propidium iodide (PI) assay was performed to assess the ability of the peptides to disrupt the cytoplasmic membrane integrity. Following membrane disruption, PI can permeate the cell membrane and intercalate with DNA, producing a fluorescent signal (Fig. 4f). After a 30-min co-incubation with PI, the results revealed that at 1 × MIC, PL-15 generated a stronger fluorescence signal compared to polymyxin B and control, indicating a more pronounced disruption of membrane integrity at this lower concentration. At 10 × MIC, polymyxin B elicited the strongest fluorescence response, followed by PL-15. Compared to polymyxin B, PL-15 demonstrated significant cytoplasmic membrane-disruptive activity that remained effective across both low and high concentration levels, with less dependence on concentration (Fig. 4g).

Additionally, the depolarization of the cytoplasmic membrane in CRAB was assessed using 3,3′-dipropylthiadicarbocyanine iodide (DiSC_3_- (5)) (Fig. 4h). After a 30-min incubation, PL-15 at 10 × MIC demonstrated the most rapid and strongest membrane depolarization, followed by polymyxin B at the same concentration. Notably, PL-15 maintained a consistently higher fluorescence intensity compared to polymyxin B throughout the observation period. At 1 × MIC, neither PL-15 nor polymyxin B induced significant depolarization, indicating a clear dose-dependent effect (Fig. 4i). These findings collectively suggest that PL-15 exerts its bactericidal effects by disrupting both the outer and cytoplasmic membranes of bacterial cells.

### PL-15 can impair bacterial energy metabolism

The disruption of bacterial membranes is known to induce excessive intracellular accumulation of ROS (52). To assess ROS generation in CRAB upon treatment with PL-15, we employed the fluorescent probe 2′,7′-dichlorodihydrofluorescein diacetate (DCFH-DA). Our results demonstrated that PL-15 elicited a concentration-dependent surge in ROS levels (Fig. 4j). Notably, pre-treatment with the ROS scavenger N-acetylcysteine (NAC) markedly diminished the antibacterial efficacy of PL-15, suggesting that oxidative stress contributes significantly to its MoA. These findings collectively indicate that, in addition to direct membrane disruption, ROS-mediated oxidative damage constitutes a critical secondary mechanism underpinning the bactericidal activity of PL-15.

### PL-15 can inhibit biofilm formation and eradicate mature biofilms

Biofilm-associated bacterial infections refer to the aggregation of bacteria on solid surfaces (such as organs, implants, or medical devices), forming a protective structure known as a biofilm. In this state, bacteria are encased in a sticky extracellular matrix that they secrete, making them more resistant to antimicrobial agents compared to planktonic bacteria. The biofilm structure impedes antibiotic penetration and enhances bacterial resistance by altering their metabolic states, which significantly complicates the treatment of such infections. Therefore, the anti-biofilm activity of antimicrobial agents is critical for their therapeutic efficacy against biofilm-associated infections (53, 54). In this study, we conducted crystal violet staining assays to evaluate potential in inhibiting and eradicating biofilms of PL-15. As shown in Fig. 4k, PL-15 and polymyxin B exhibited moderate biofilm inhibition at 1/2 × MIC, with their effectiveness progressively increasing with higher peptide concentrations. In terms of biofilm eradication, both PL-15 and polymyxin B demonstrated comparable levels of biofilm inhibition (Fig. 4l). The inhibition of biofilm formation is likely related to its membrane-disrupting and bactericidal properties, while the eradication of mature biofilms may result from rapid killing of biofilm-embedded bacteria, leading to structural collapse. Although PL-15 is not a typical quorum sensing inhibitor, its anti-biofilm effects may partially involve the downregulation of signaling pathways such as ppGpp (55). This finding underscores the dual-action potential of PL-15, making it a promising therapeutic agent not only for planktonic bacterial infections but also for biofilm-associated infections, a critical challenge in clinical treatment.

### PL-15 shows low propensity to induce resistance

The growing issue of antibiotic resistance has prompted us to conduct a 20-passage resistance induction experiment on CRAB (Fig. 4m). After serial passaging at sub-MIC concentrations of PL-15 for 20 days, no detectable resistance emerged, with MIC fluctuations remaining within a twofold range. In contrast, polymyxin B exhibited a substantial increase in resistance, with the MIC elevated by 64-fold under the same conditions. These results indicate that PL-15 possesses a much lower tendency to induce resistance compared to polymyxin B, highlighting its favorable resistance profile. This low propensity for resistance development may be attributed to PL-15’s potent bactericidal activity, coupled with its ability to inhibit biofilm formation and eradicate mature biofilms, properties that further enhance its therapeutic potential.

## DISCUSSION

In this study, we presented a novel approach for discovering AMPs based on LLMs. By leveraging AMPGenix-generated peptides as foundational templates, we were able to rapidly identify several novel peptides exhibiting significant antimicrobial activity against a broad range of clinical MDR pathogens, including CRAB and MRSA. In particular, PL-15 demonstrated superior broad-spectrum antimicrobial activity compared to k_AMPs, while maintaining low cytotoxicity. As a bactericidal agent, PL-15 achieved rapid bacterial killing at low concentrations and showed remarkable therapeutic efficacy against CRAB infections *in vivo*. MoA results showed that PL-15 exerts its bactericidal effects through disrupting outer membrane stability, impairing inner membrane integrity, inducting cytoplasmic membrane depolarization, and elevating intracellular ROS levels, which leads to rapid bacterial cell death. Meanwhile, PL-15 demonstrated dual antibiofilm capabilities, effectively inhibiting biofilm formation and eradicating pre-established mature biofilms, which reduces the propensity for resistance development. Subsequent studies will focus on enhancing the biocompatibility and safety of PL-15 through rational amino acid substitutions. In addition, efforts will be made to identify the minimal functional sequence required for antimicrobial activity, which would allow for peptide truncation and reduced production costs. These strategies aim to improve the therapeutic potential of PL-15 and facilitate its large-scale production and clinical translation.

Utilizing generated peptides as templates for the development of derivative peptide analogs offers an opportunity to assess whether the generative model effectively captures the biological patterns characteristic of AMPs. This approach also facilitates the evaluation of the model’s reliability and stability, particularly under non-traditional conditions. It is important to highlight that the introduction of generative model provided a superior framework for AMP design through sequence alignment. By leveraging transfer learning, AMPGenix was capable of capturing the unique amino acid composition and distribution patterns inherent in AMP sequences, thereby providing robust prior knowledge for AMP design. This prior knowledge aided in defining the core structural features of AMPs and made the design process more systematic and structured. In comparison to traditional methods that rely on manual design and peptide modification (43, 56, 57), the strategy combining generative model with sequence alignment significantly enhanced the development efficiency of functional substances. Traditional approaches often depend on empirical rules and repeated validation, which are typically constrained by high costs and prolonged timeframes, and limited exploration of sequence diversity. In contrast, the “generative-alignment” approach seamlessly integrates artificial intelligence-based generation and data mining, seamlessly combining these methods to optimize the design process. By leveraging prior knowledge obtained through transfer learning in the upstream phase, the generative model automatically identifies and generates peptide sequences that meet specific functional requirements. In the downstream phase, sequence alignment techniques are employed to rapidly uncover uncharacterized “dark matter” embedded within proteome, including encrypted peptides concealed within the broader sequences of functional proteins. This strategy not only facilitated the rapid expansion of the candidate pool but also effectively preserved functional characteristics, enabling the discovery of novel analogs with enhanced properties.

## MATERIALS AND METHODS

### Sequence alignment

The sequence alignment was performed using the template WLKKILKWLKW with the online tool available at https://blast.ncbi.nlm.nih.gov/Blast.cgi.

### Structure and property prediction

The structures of proteins (Fig. 1a) and peptides (Fig. 1c) were predicted by AlphaFold server (https://alphafoldserver.com/), while molecular weight and net charge in pH 7.4 of c_AMPs were predicted using Isoelectric Point Calculator 2.0 (https://ipc2.mimuw.edu.pl/).

### Peptide

Peptides with more than 95% purity were custom synthesized by SynthBiological Engineering Co., Ltd. (Hefei, China). Each peptide was aliquoted into multiple tubes at 2 mg per tube and stored at −80°C. The peptide powder was dissolved on the day of the experiment and diluted to the appropriate concentration for use.

### Chemicals

Luria Bertani (LB) agar, LB broth, Mueller-Hinton broth (MHB), and MacConkey agar were purchased from Hopebiol (Qingdao, China). Cell Counting Kit-8 was purchased from MedChemExpress (Shanghai, China). Dulbecco’s Modified Eagle Medium/high glucose medium, penicillin-streptomycin mixed solution (dual antibody), fetal bovine serum, and trypsin/EDTA solution were purchased from Shanghai Zhong Qiao Xin Zhou Biotechnology Co., Ltd. (Shanghai, China). Triton X-100 was purchased from Sigma-Aldrich (St. Louis, MO, USA). Dimethylsulfoxide (DMSO), cyclophosphamide, vancomycin, and DiSC_3_- (5) were purchased from Mcklin (Shanghai, China). PBS, HEPES, and PI were obtained from Solarbio (Beijing, China). D-Glucopyranose and potassium chloride (KCl) were obtained from Hushi (Shanghai, China). NPN was obtained from Beyotime Biotechnology (Shanghai, China).

### Bacterial strains and growth conditions

Laboratory-maintained *E. coli* ATCC25922, *S. aureus* ATCC25923, *A. baumannii* ATCC19606, and five clinical isolates (CRAB 2210186, MRSA 2210187, CRE 2403262, CRPA 2403282, and CRKP 2403276) were used. All strains were grown and plated on LB at 37°C.

### Mice

All animal experiments were performed according to the “Principles of Laboratory Animal Care” (NIH publication No. 86-23, revised 1985) and approved by the Animal Care and Use Committee of Shandong University (LL20240622, Jinan, China). Five-week-old male BALB/c mice were purchased from Beijing Vital River Laboratory Animal Technology Co., Ltd. (Beijing, China) and kept under a 12-hour light/12-hour dark cycle, humidity of 50%, and temperature of 22°C in standard specific pathogen-free individually vented cages.

### Antimicrobial activity

The MIC of all peptides was determined using the microdilution broth method in a 96-well plate. Peptides were dissolved in the DMSO with an initial concentration of 12,800 µg mL^−1^. And cells of eight strains were separately suspended in the MHB at a density of approximately 10^5^ CFU mL^−1^. In the first column of the 96-well plate, 16 µL of peptide and 184 µL of MHB were added to each well to achieve a final concentration of 1,024 µg mL^−1^. One-hundred microliters of MHB was added to each of the remaining wells. Then the first column of liquid was mixed well and diluted to the 12th column using the twofold dilution method. One-hundred microliters of prepared bacterial solution was added to all wells, and the 96-well plate was incubated at 37°C for 18–24 h. The MIC was defined as the concentration of the peptide in the well just preceding the first well showing visible turbidity. All experiments were performed in triplicate to ensure reliability.

### Hemolysis test

Freshly collected sheep red blood cells, provided by Solarbio (Beijing, China), were washed three times with PBS to prepare a red blood cell suspension (2,000 rpm). Subsequently, the resulting red blood cells were suspended in 1 × PBS buffer and adjusted to a 4% red blood cell suspension. In a 96-well plate, perform twofold serial dilutions of the test peptides in 1 × PBS to final concentrations of 512, 256, 128, 64, 32, 16, 8, 4, 2, 1, 0.5, and 0.25 µg mL^−1^ in each well. Use Triton X-100 as a positive control and 1 × PBS buffer as a negative control. Take 100 µL of red blood cell solution and 100 µL of peptide solutions with different concentration gradients and control solutions, add them to a new 96-well plate, and incubate at 37°C for 1 h. Subsequently, the 96-well plate was centrifuged at 4,000 rpm for 10 min, and the supernatant was collected into a 96-well plate for later use. Then the absorbance was measured at a wavelength of 450 nm using a microplate reader. All experiments were repeated in three parallel groups and averaged. The hemolysis rate was calculated by the following formula and the IC50 value of each AMPs was calculated by online tools: https://www.aatbio.com/tools/ic50-calculator.


(1) $$\begin{array}{c} Hemolysis\;rate\;\left(\%\right)=\frac{OD\left(AMP\right)-\;OD\left(PBS\right)}{OD\left(TritonX-100\right)-\;OD\left(PBS\right)}\times 100\% \end{array}$$

### Cytotoxicity against mammalian cells

Cytotoxicity of AMPs was determined using the Cell Counting Kit-8. HEK293 cells, bought from Procell (Wuhan, China), were inoculated in 96-well flat-bottom plates according to 5,000 cells/well in cell culture medium. After 24-h incubation at 37°C with 5% CO_2_ in atmosphere, medium was replaced with fresh medium and incubated with antimicrobial peptide solutions at various concentrations for 48 h. Cell viability was monitored by adding CCK-8 solution and measuring OD_450_ after 4 h. Zero-well and control-well were set during this experimental process. All experiments were performed with three independent replicates. The cell survival rate was calculated by the following formula, and the CC50 value of each AMPs was calculated by online tools: https://www.aatbio.com/tools/ic50-calculator.


(2) $$\begin{array}{c} Cell\;survival\;rate\;\left(\%\right)=\frac{OD\left(AMP\right)-\;OD\left(zero\right)}{OD\left(control\right)-\;OD\left(zero\right)}\times 100\% \end{array}$$

### MBC determination

The MBC was determined using the microdilution broth method described above for MIC determination. After identifying the clear wells (no growth), 10 µL of the liquid from these wells was transferred and spread on a nutrient agar plate. The plate was incubated at 37°C for 18–24 h. After incubation, the surface of the plate was observed for bacterial colony growth. The lowest concentration with no visible growth was considered the MBC.

### Time-kill curve

Bacteria in the logarithmic growth phase were diluted to approximately 10^5^ CFU mL^−1^ in MH broth and treated with peptides, polymyxin B, or vancomycin at final concentrations of 1/2 × MIC, 1 × MIC, 2 × MIC, and 4 × MIC. After the addition of the test compound, samples were taken at 0, 3, 6, 9, 12, and 24 h and serially diluted. Subsequently, 5 µL of the diluted samples were spread on agar plates and incubated at 37°C for 20 h. The bacterial concentration CFU mL^−1^ at the corresponding time point of each sample was calculated based on the number of colonies on the agar plate, and the log_10_ (CFU mL^−1^) was plotted against time (h).

### Serum sensitivity testing

For serum sensitivity experiments, the peptide to be tested was incubated in 10% fetal bovine serum at room temperature for 1–6 h. After deactivation, the MICs of peptides against CRAB 2210186 and MRSA 2210187 were measured using the MIC assay. All experiments were performed in triplicate to ensure reliability.

### Thigh infection mouse model

To establish the mouse thigh infection model, mice were injected intraperitoneally with cyclophosphamide 4 d and 1 d before bacterial administration, at concentrations of 150 and 100 mg kg^−1^, respectively, to induce neutropenia (51). CRAB 2210186 cells were suspended in sterile PBS, adjusted to a concentration of ~1  ×  10^6^ CFU per infection site and injected into the right thighs of eight mice per treatment group. PL-15 (10 × MIC, 100 µL), polymyxin B (20,000–25,000 U kg^−1^ day^−1^) or PBS were administered intraperitoneally at 1, 3, 5, and 7 h post-infection. Mice were euthanized 24 h after infection, and the thigh tissues were aseptically collected, weighed, homogenized, serially diluted in PBS, and plated onto solid MCA. The CFU of CRAB 2210186 per thigh was calculated by plating dilutions of the thigh homogenates (0.25 g of thigh tissue in 10 mL sterile PBS) on agar plates and counting colonies.

### MD simulations

To investigate the interactions between peptides and bacterial membranes, all-atom MD simulations were performed using GROMACS 2024.5 (www.gromacs.org) (58). The simulation systems were constructed using CHARMM-GUI Membrane Builder (59), and the 3D structure of each peptide was predicted using AlphaFold2 (60). The bilayer membrane system consisted of 120 molecules of POPE and 60 molecules of POPG. The peptide was initially placed 3 nm above the membrane surface. The system was solvated with water containing K^+^ and Cl⁻ ions, with ion concentrations adjusted to 0.15 mol L^−1^ to neutralize the total system charge. All peptides, lipids, and ions were modeled using the CHARMM36m force field (61), and water molecules were represented by the TIP3P water model (62). Electrostatic interactions were calculated using the Particle Mesh Ewald method, and van der Waals interactions were truncated at 12 Å. All bonds involving hydrogen atoms were constrained using the LINCS algorithm (62).

The system first underwent 5,000 steps of energy minimization using the steepest descent method. This was followed by six equilibration steps, during which the positional restraints on peptides and lipids were gradually removed. The velocity Verlet integrator was used during equilibration. V-rescale temperature coupling and C-rescale pressure coupling were applied. Finally, unrestrained production MD simulations were conducted at 310 K and 1 bar using a Nose-Hoover thermostat and Parrinello-Rahman barostat. The time step was two fs, and the total simulation time was 200 ns for PL-15.

### SEM measurement

SEM measurement was performed following previous report (51). CRAB 2210186 was grown to the exponential phase at 37°C overnight. The bacterial suspensions (10^8^ CFU mL^−1^) were co-cultured with AMPs at final concentrations of one- and 10-fold MIC at 37°C for 24 h, and untreated cells were used as control. Samples were washed with PBS three times and treated with glutaraldehyde for 4 h at 4°C. Sample (10  µL) was added onto a silicon wafer and sequentially dehydrated with 30%, 60%, 90%, 95%, and 100% ethanol/water (10 min each). The specimens were observed using SEM (FE-SEM Regulus8100, Hitachi).

### PI assay

PI (which is known to stain nuclear chromatin upon cell membrane disruption) assay was carried out to evaluate the ability of AMPs to disrupt the cytoplasmic membrane integrity (63). After CRAB was grown to the exponential phase, bacteria cells were harvested and washed three times in washing buffer of 5 mM glucose and 5 mM HEPES (1:1, pH = 7.2) and then re-adjusted to 10^8^ CFU mL^−1^ in the same buffer. Bacterial suspensions (150 µL/well) were loaded into black-walled, clear-bottom 96-well plates with lids. Following bacterial loading, 50 µL of 10 µM PI was introduced into each well, with 30 min pre-incubation CRAB. Post-incubation fluorescence signals were acquired using 535 nm excitation and 617 nm emission wavelengths. Compounds (10 µL) were then administered to achieve 1 × MIC/10 × MIC-equivalent final concentrations in the dye-loaded bacterial suspensions. Real-time fluorescence monitoring was performed with continuous acquisition during the first 10 min, followed by measurements at 10-min intervals. Negative controls received 10 µL sterile water in lieu of antimicrobial peptides while maintaining equivalent pre-incubation conditions. All experimental conditions were executed with three biological replicates.

### Cytoplasmic membrane depolarization assay

DiSC_3_- (5) accumulates in the cytoplasmic membrane and self-quenches its own fluorescence; however, when membrane potential is disrupted, DiSC_3_- (5) is released into the culture media (64). DiSC_3_- (5) assay was performed to evaluate the ability of AMPs to depolarize bacterial cytoplasmic membrane. After CRAB was grown to the exponential phase, bacteria cells were harvested and washed three times in washing buffer of 5 mM glucose and 5 mM HEPES (1:1, pH = 7.2) and then re-adjusted to 10^8^ CFU mL^−1^ in buffer of 5 mM glucose, 5 mM HEPES, and 100 mM KCl (1:1:1). Bacterial suspensions (100 µL/well) were loaded into black-walled, clear-bottom 96-well plates with lids. Following bacterial loading, 50 µL of 8 µM DiSC_3_- (5) and 50 µL of 200 µM EDTA were introduced into each well, with 30-min pre-incubation CRAB. Post-incubation fluorescence signals were acquired using 622 nm excitation and 670 nm emission wavelengths. Compounds (10 µL) were then administered to achieve 1 × MIC/10 × MIC-equivalent final concentrations in the dye-loaded bacterial suspensions. Real-time fluorescence monitoring was performed with continuous acquisition during the first 10 min, followed by measurements at 10-min intervals. Negative controls received 10 µL sterile water in lieu of antimicrobial peptides while maintaining equivalent pre-incubation conditions. All experimental conditions were executed with three biological replicates.

### Outer membrane permeability assay

NPN binds phospholipids in the periplasmic space, so NPN assay was carried out to analyze the effect of AMPs on the outer membrane permeabilization of bacteria (65). After CRAB was grown to the exponential phase, bacteria cells were harvested and washed three times in washing buffer of 5 mM glucose and 5 mM HEPES (1:1, pH = 7.2) and then re-adjusted to 10^8^ CFU mL^−1^ in same buffer. Bacterial suspensions (150 µL/well) were loaded into black-walled, clear-bottom 96-well plates with lids. Following bacterial loading, 50 µL of 10 µM NPN was introduced into each well, with 30-min pre-incubation for CRAB. Post-incubation fluorescence signals were acquired using 350 nm excitation and 420 nm emission wavelengths. Compounds (10 µL) were then administered to achieve 1 × MIC/10 × MIC -equivalent final concentrations in the dye-loaded bacterial suspensions. Real-time fluorescence monitoring was performed with continuous acquisition during the first 10 min, followed by measurements at 10-min intervals. Negative controls received 10 µL sterile water in lieu of antimicrobial peptides while maintaining equivalent pre-incubation conditions. All experimental conditions were executed with three biological replicates.

### ROS accumulation assay

Bacteria in the logarithmic growth phase were prepared to OD600 = 0.5. Ten microliters of DCFH-DA was added to 190 µL of bacterial solution to a final concentration of 10 µM and incubated at 37°C for 30 min. After washing with PBS three times, 190 µL of probe-labeled bacterial cells were added to a 96-well plate, and 10 µL of the sample to be tested was added. After incubation for another 2 h, the fluorescence intensity was immediately measured using a microplate reader with an excitation wavelength of 488 nm and an emission wavelength of 525 nm. The antioxidant NAC (6 mM) was used as a control to neutralize the production of ROS.

### Biofilm formation inhibition assay

The crystal violet staining method (66) was used to determine the inhibitory activity of the compounds against the biofilm formation of CRAB. The steps were as follows: prepare a bacterial solution (10^6^ CFU mL^−1^), mix it with different final concentrations of the compound (1/2 × MIC, 1 × MIC, 2 × MIC, and 4 × MIC), and culture it for 24 h. After discarding the supernatant, wash with PBS, add anhydrous methanol to fix the biofilm, and then stain it with crystal violet for 60 min after drying. After washing, dissolve the biofilm with 33% glacial acetic acid, and measure OD570. Three parallel experiments were performed, and the biofilm formation inhibition rate was calculated by the following formula:


(3) $$\begin{array}{c} Biofilm\;formation\;inhibition\;rate\;\left(\%\right)=\left(1-\frac{OD\left(sample\right)-\;OD\left(blank\right)}{OD\left(negative\right)-\;OD\left(blank\right)}\right)\times 100\% \end{array}$$

### Mature biofilm eradication experiment

Bacteria in the logarithmic growth phase were diluted with LB broth to OD620 = 0.05. After continuing to culture the bacteria in the logarithmic growth phase, the bacterial solution was diluted to OD620 = 0.05 and set aside. One-hundred microliters of the bacterial solution was added to a 96-well plate and placed in a 37°C incubator for 24 h; the liquid in the well was discarded, and the well was carefully washed twice with PBS, and then the sample diluted with PBS was added and incubated for 24 h. The subsequent treatment procedure was the same as the biofilm formation inhibition experiment.

### Drug resistance assay

Resistance development assays were performed following previous report (43). CRAB was cultured overnight at 37°C at 160 rpm in LB. In wells of a 96-well polypropylene flat-bottom plate, 5 µL of the overnight bacterial culture (10^7^ CFU mL^−1^) was added to 100 µL of peptides/antibiotic solutions (with final concentrations of 0.25 to 512 µg mL^−1^) in MHB. Plates were incubated for 18 to 20 h at 37°C. The MIC, the lowest concentration of peptides/antibiotic that caused lack of visible bacterial growth, was determined for each bacterial species. Thereafter, 5 µL of the growth at the 0.5-fold MIC suspension (10^7^ CFU mL^−1^) was added to a fresh medium containing peptides/antibiotic, and these mixtures were incubated as described above. This was repeated for 20 passages. All experiments were performed in triplicate to ensure reliability.

## Data Availability

The original contributions presented in this study are included in the article/supplementary material. Further inquiries can be directed to the corresponding author(s).

## SUPPLEMENTAL MATERIAL

Table S1 spectrum.01504-25-s0001.xlsx The BLAST output of top five protein sequences with the highest Max Scores.

Table S2 spectrum.01504-25-s0002.xlsx Structure and mechanism of action of 47 known AMPs.

ASM does not own the copyrights to Supplemental Material that may be linked to, or accessed through, an article. The authors have granted ASM a non-exclusive, world-wide license to publish the Supplemental Material files. Please contact the corresponding author directly for reuse.
